# An Updated Survey on Statistical Thresholding and Sample Size of fMRI Studies

**DOI:** 10.3389/fnhum.2018.00016

**Published:** 2018-01-26

**Authors:** Andy W. K. Yeung

**Affiliations:** Oral and Maxillofacial Radiology, Applied Oral Sciences, Faculty of Dentistry, The University of Hong Kong, Pok Fu Lam, Hong Kong

**Keywords:** false-discovery rate, familywise error rate, fMRI, Gaussian random field, literature, Monte Carlo stimulation, threshold, threshold-free cluster enhancement

## Abstract

**Background:** Since the early 2010s, the neuroimaging field has paid more attention to the issue of false positives. Several journals have issued guidelines regarding statistical thresholds. Three papers have reported the statistical analysis of the thresholds used in fMRI literature, but they were published at least 3 years ago and surveyed papers published during 2007–2012. This study revisited this topic to evaluate the changes in this field.

**Methods:** The PubMed database was searched to identify the task-based (not resting-state) fMRI papers published in 2017 and record their sample sizes, inferential methods (e.g., voxelwise or clusterwise), theoretical methods (e.g., parametric or non-parametric), significance level, cluster-defining primary threshold (CDT), volume of analysis (whole brain or region of interest) and software used.

**Results:** The majority (95.6%) of the 388 analyzed articles reported statistics corrected for multiple comparisons. A large proportion (69.6%) of the 388 articles reported main results by clusterwise inference. The analyzed articles mostly used software Statistical Parametric Mapping (SPM), Analysis of Functional NeuroImages (AFNI), or FMRIB Software Library (FSL) to conduct statistical analysis. There were 70.9%, 37.6%, and 23.1% of SPM, AFNI, and FSL studies, respectively, that used a CDT of *p* ≤ 0.001. The statistical sample size across the articles ranged between 7 and 1,299 with a median of 33. Sample size did not significantly correlate with the level of statistical threshold.

**Conclusion:** There were still around 53% (142/270) studies using clusterwise inference that chose a more liberal CDT than *p* = 0.001 (*n* = 121) or did not report their CDT (*n* = 21), down from around 61% reported by Woo et al. (2014). For FSL studies, it seemed that the CDT practice had no improvement since the survey by Woo et al. (2014). A few studies chose unconventional CDT such as *p* = 0.0125 or 0.004. Such practice might create an impression that the threshold alterations were attempted to show “desired” clusters. The median sample size used in the analyzed articles was similar to those reported in previous surveys. In conclusion, there seemed to be no change in the statistical practice compared to the early 2010s.

## Introduction

Functional magnetic resonance imaging (fMRI) studies—particularly the task-based fMRI studies, the most popular type of fMRI study—enable researchers to examine the human brain about various aspects ranging from sensation to cognition. Findings may bear clinical relevance such as the identification of neural correlates of diseases or the enabling of a neuro-functional assessment of clinical treatments.

The reproducibility of a neuroscience report depends on numerous factors—including the methodological details, statistical power and flexibility of the analyses (Carp, 2012). One of the most important factors that could be assessed relatively easily is the statistical approach used. Every paper may set its own significance level for the statistical tests reported (Hupé, 2015), and therefore, one may need to interpret the significant results from different papers differently. Considering the mass-univariate analytic approach utilized by various popular fMRI data-processing software—such as Statistical Parametric Mapping (SPM) (Penny et al., 2011), Analysis of Functional NeuroImages (AFNI) (Cox, 1996), and FMRIB Software Library (FSL) (Jenkinson et al., 2012) —correction for multiple comparisons is crucial for simultaneous statistical tests on several thousands of voxels. With regard to proper corrections for multiple comparisons, Carp (2012) revealed that an astonishing 41% of his 241 surveyed studies, which were published during 2007–2012, did not report formal corrections. As an extension to his work, Guo et al. (2014) reported a much reduced 19% for their 100 surveyed studies, which were published in six leading neuroscience/neuroimaging/multidisciplinary journals during 2010–2011. Similarly, Woo et al. (2014) reported that 6% of their 814 surveyed studies, which were published in seven leading journals during 2010–2011, did not apply formal statistical corrections. Uncorrected results may contain high false-positive rates, and therefore, their reproducibility and clinical relevance could potentially be undermined. Even for corrected results, the improper setting of statistical thresholds may also lead to inflated false-positive rates. Woo et al. (2014) and Eklund et al. (2016) have repeatedly stated that routine voxelwise correction methods are adequate for controlling false positives whereas cluster-defining primary thresholds (CDT) for clusterwise inferences should be set at *p* = 0.001 or lower because more liberal thresholds, such as *p* = 0.01, may cause highly inflated false-positive rates for parametric methods. Clusterwise inference was the most popular method because it is more sensitive when detecting significance (i.e., more powerful); however, its spatial precision is inferior to that of voxelwise inference, as a large significant cluster can only indicate that significant activations are contained within the cluster. Clusterwise inference gives no information with regard to which voxels are significantly activated (Woo et al., 2014).

In 2016, two journals issued guidelines regarding their stance on the standard statistical thresholds of reported fMRI/neuroimaging results (Carter et al., 2016; Roiser et al., 2016). **Table 1** lists the key points of these guidelines and the suggestions of Woo et al. (2014) and Eklund et al. (2016). Moreover, several years have lapsed since 2014, the year when the last survey was published (Guo et al., 2014). It is time to conduct a literature survey on the statistical thresholds used by the fMRI studies published most recently.

**Table 1:** Recently published recommended statistical practices for controlling false positives.

Publication name	Recommendations
Woo et al., 2014	1. Set the default cluster-defining primary threshold (CDT) at *p* < 0.001. 2. Use a stringent CDT or voxelwise inference for highly powered studies.
Eklund et al., 2016	1. The parametric method works well for voxelwise inferences but not for clusterwise inferences (unless a stringent CDT is set at *p* < 0.001). 2. The permutation method works well for both voxelwise and clusterwise inferences.
Roiser et al., 2016	1. For clusterwise inferences, choose a stringent CDT (e.g., *p* < 0.001) unless the permutation method was employed. 2. For voxelwise inferences, *p*-values should be corrected for multiple comparisons. 3. Complementary approaches, such as false-discovery rate or threshold-free cluster enhancement, can be considered. 4. Preregister the proposed studies in which the planned statistical analyses methods are documented clearly.
Carter et al., 2016	1. Studies investigating very small brain regions should use a high voxel threshold (e.g., *p* < 0.001). 2. Studies not targeting precise localization may consider a more liberal threshold and focus on controlling false negatives by data reduction (e.g., region-of-interest analyses), as studies with fewer than 50 subjects per group usually have limited power.

## Materials and Methods

In accordance with the methods of previous studies (Carp, 2012; Guo et al., 2014), articles published in 2017 and written in English were identified with the keywords “fMRI,” “BOLD,” and “task” in the PubMed database. The search was performed on July 20, 2017. These criteria yielded 1,020 articles (listed in **Supplementary File S1**). For this study, all 1,020 articles were initially included, and each was assessed by reading its full text and excluded if it did not report task-based human fMRI studies and did not report results from SPM. In other words, studies that reported animal studies, resting-state fMRI, connectivity, multi-voxel pattern analysis or percent of signal change were excluded. The screening excluded 632 articles accordingly and finally a total of 388 articles entered the analysis (**Supplementary File S1**). For the 388 articles, items including sample size, inferential method (e.g., voxelwise or clusterwise), theoretical method of correction for multiple comparisons (e.g., parametric or non-parametric), significance level, CDT (if applicable), volume of analysis (whole brain or region of interest; ROI) and software used were recorded manually. For articles that used multiple thresholds, the most stringent one used for the main analyses was chosen (Woo et al., 2014). Pearson’s correlation test was performed to evaluate the relationship between the sample size and the levels of CDT in the articles using clusterwise inference.

## Results

### Sample Size and Software Used

**FIGURE 1:**
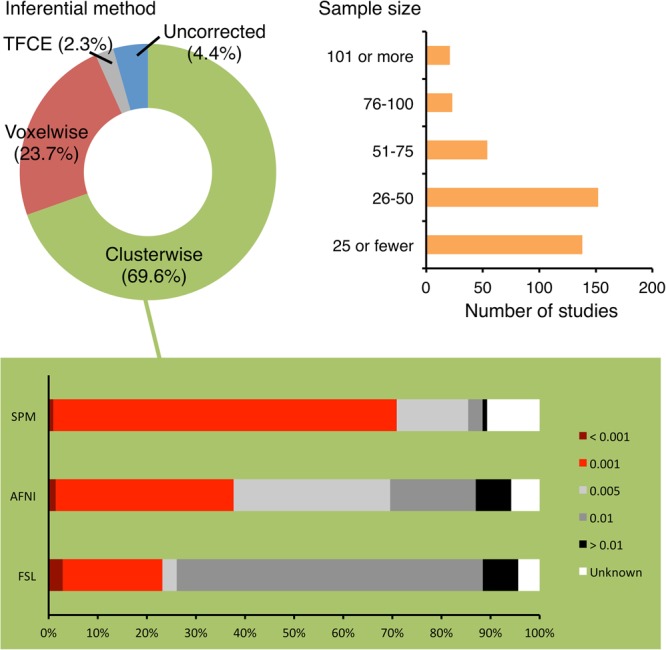
Choices of inferential methods and sample sizes used by the surveyed studies. The majority of the surveyed studies used clusterwise inference and recruited 50 subjects or fewer. For the studies using clusterwise inference, the cluster-defining primary thresholds (CDTs) used by them were recorded. According to Woo et al. (2014) and Eklund et al. (2016), a CDT at or more stringent than *p* = 0.001 is recommended (indicated by red portions of the bars in the lower panel). This was achieved by 70.9%, 37.6%, and 23.1% of studies using SPM, AFNI, and FSL, respectively.

The sample size reported in 388 papers ranged from 7 to 1,299 with a median of 33. One hundred and thirty-eight studies (35.6%) analyzed data from 25 or fewer subjects, 152 studies (39.2%) had 26–50 subjects, 54 studies (13.9%) had 51–75 subjects, 23 studies (5.9%) had 76–100 subjects and 21 studies (5.4%) had 101 or more subjects (**Figure 1**).

The studies were published in 125 journals (**Table 2**). The studies predominantly used SPM for statistical analyses (202, 52.1%)—followed by FSL (79, 20.4%), AFNI (71, 18.3%), BrainVoyager (11, 2.8%), Resting-State fMRI Data Analysis Toolkit (6, 1.5%), Statistical Non-Parametric Mapping (SnPM; 5, 1.3%), and Matlab but other toolbox than SPM or SnPM (5, 1.3%). There was one study that used FreeSurfer, one used MAsks for Region of INterest Analysis, one used FIDL (developed by Washington University in St. Louis), one used TFCE toolbox (University of Jena) and one used XBAM (developed by King’s College London).

**Table 2:** The 125 journals that published the 388 analyzed articles.

Journal list	Count	%	Journal list (continued)	Count	%
Neuroimage	23	5.9	Alzheimers Dement (Amst)	1	0.3
Cortex	14	3.6	Appl Neuropsychol Child	1	0.3
Neuropsychologia	14	3.6	Arch Gerontol Geriatr	1	0.3
Brain Imaging Behav	13	3.4	Behav Res Ther	1	0.3
Cereb Cortex	13	3.4	BMC Psychiatry	1	0.3
Hum Brain Mapp	13	3.4	Br J Psychiatry	1	0.3
J Neurosci	12	3.1	Br J Sports Med	1	0.3
PloS One	12	3.1	Cerebellum	1	0.3
Sci Rep	12	3.1	Cogn Neurosci	1	0.3
J Cogn Neurosci	11	2.8	Cultur Divers Ethnic Minor Psychol	1	0.3
Behav Brain Res	10	2.6	Dev Psychol	1	0.3
Psychiatry Res	9	2.3	Einstein (Sao Paulo)	1	0.3
J Affect Disord	8	2.1	Emotion	1	0.3
Neuroimage Clin	8	2.1	Epilepsy Behav	1	0.3
Brain Struct Funct	6	1.5	Eur Child Adolesc Psychiatry	1	0.3
Neuroscience	6	1.5	Eur Eat Disord Rev	1	0.3
Soc Cogn Affect Neurosci	6	1.5	Eur J Paediatr Neurol	1	0.3
Addict Biol	5	1.3	Eur J Pain	1	0.3
Biol Psychol	5	1.3	Eur Neuropsychopharmacol	1	0.3
Dev Psychopathol	5	1.3	Eur Radiol	1	0.3
Neuropsychopharmacology	5	1.3	Front Aging Neurosci	1	0.3
Psychol Med	5	1.3	Front Neuroanat	1	0.3
Soc Neurosci	5	1.3	Front Psychol	1	0.3
Addiction	4	1	Int J Neuropsychopharmacol	1	0.3
Brain Cogn	4	1	Int J Neurosci	1	0.3
Brain Res	4	1	Int J Psychophysiol	1	0.3
Cogn Affect Behav Neurosci	4	1	J Alzheimers Dis	1	0.3
Dev Sci	4	1	J Am Acad Child Adolesc Psychiatry	1	0.3
Eur J Neurosci	4	1	J Atten Disord	1	0.3
Front Behav Neurosci	4	1	J Autism Dev Disord	1	0.3
Front Hum Neurosci	4	1	J Child Sex Abus	1	0.3
Mult Scler	4	1	J Clin Exp Neuropsychol	1	0.3
Transl Psychiatry	4	1	J Hypertens	1	0.3
Biol Psychiatry	3	0.8	J Neurol Neurosurg Psychiatry	1	0.3
Brain Behav	3	0.8	J Neuropsychol	1	0.3
Brain Lang	3	0.8	J Neurotrauma	1	0.3
Elife	3	0.8	J Orthop Sports Phys Ther	1	0.3
Eur Arch Psychiatry Clin Neurosci	3	0.8	J Physiol Anthropol	1	0.3
Neural Plast	3	0.8	J Psycholinguist Res	1	0.3
Psychopharmacology (Berl)	3	0.8	J Speech Lang Hear Res	1	0.3
Schizophr Res	3	0.8	J Vis Exp	1	0.3
Alcohol Alcohol	2	0.5	J Voice	1	0.3
Am J Psychiatry	2	0.5	JAMA Psychiatry	1	0.3
Bipolar Disord	2	0.5	Nat Commun	1	0.3
Brain Stimul	2	0.5	Neural Regen Res	1	0.3
Brain Topogr	2	0.5	Neurobiol Learn Mem	1	0.3
Brain	2	0.5	Neurodegener Dis	1	0.3
Clin Physiol Funct Imaging	2	0.5	Neurogastroenterol Motil	1	0.3
Depress Anxiety	2	0.5	Neurol Med Chir (Tokyo)	1	0.3
Dev Cogn Neurosci	2	0.5	Neurology	1	0.3
Drug Alcohol Depend	2	0.5	Neuropsychobiology	1	0.3
Exp Brain Res	2	0.5	Neuroradiology	1	0.3
Hippocampus	2	0.5	Nutr Neurosci	1	0.3
J Int Neuropsychol Soc	2	0.5	Obes Res Clin Pract	1	0.3
J Psychiatr Res	2	0.5	Physiol Rep	1	0.3
J Psychopharmacol	2	0.5	PLoS Biol	1	0.3
Mol Psychiatry	2	0.5	Proc IEEE Inst Electr Electron Eng	1	0.3
Neurobiol Aging	2	0.5	Psychiatry Clin Neurosci	1	0.3
Proc Natl Acad Sci USA	2	0.5	Psychophysiology	1	0.3
Prog Neuropsychopharmacol Biol Psychiatry	2	0.5	Res Dev Disabil	1	0.3
Psychoneuroendocrinology	2	0.5	Schizophr Bull	1	0.3
Acta Radiol	1	0.3	Swiss Med Wkly	1	0.3
Alcohol Clin Exp Res	1	0.3			

### Choice of Inferential Method, Theoretical Method, and Significance Level

The majority of studies (371, 95.6%) reported main results with statistics corrected for multiple comparisons. Of the analyzed studies, 270 (69.6%) reported clusterwise inference for their main analyses whereas 92 (23.7%) reported using voxelwise inference and nine (2.3%) reported using the threshold-free cluster enhancement (TFCE) inference (**Figure 1**). Most of the studies defined significance at corrected *p* = 0.05. There were 338 studies (87.1%) that reported whole-brain results for their main analyses and 244 of them (72.2%) used clusterwise inference (**Table 3**). Fifty studies (12.9%) reported ROI results and 17 studies (4.4%) reported uncorrected statistics.

**Table 3:** Thresholds of statistical significance used by the 338 surveyed studies reporting whole brain results.

Inferential method	*n*	%
Cluster-level inference (*n* = 244)		
Corrected *p* = 0.05	228	93.4
Corrected *p* = 0.025	1	0.4
Corrected *p* = 0.01	8	3.3
Corrected *p* = 0.001	7	2.9
		
Voxel-level inference (*n* = 71)		
Corrected *p* = 0.05	67	94.4
Corrected *p* = 0.025	1	1.4
Corrected *p* = 0.01	1	1.4
Corrected *p* = 0.005	1	1.4
Corrected *p* = 0.001	1	1.4
		
Threshold free cluster enhancement (*n* = 7)		
Corrected *p* = 0.05	7	100.0
		
Uncorrected inference (*n* = 16)		
*p* = 0.05, *k* = 40	1	6.3
*p* = 0.005, *k* = 50	1	6.3
*p* = 0.005, *k* = 20	1	6.3
*p* = 0.005, *k* = 10	1	6.3
*p* = 0.005	1	6.3
*p* = 0.001, *k* = 20	4	25.0
*p* = 0.001, *k* = 15	1	6.3
*p* = 0.001, *k* = 10	3	18.8
*p* = 0.001, *k* = 5	1	6.3
*p* = 0.001	2	12.5

*k means the minimal cluster size expressed in number of voxels.*

Corrections for multiple comparisons were achieved by various theoretical methods (**Table 4**)—predominantly parametric methods, regardless of inference at cluster or voxel level. Five studies did not mention their theoretical methods, and all of them used FSL software.

**Table 4:** Cross-tabulation of the theoretical methods and statistical thresholds of the 371 surveyed studies reporting corrected statistics.

Inferential method	Theoretical method					Total count
	Parametric (FWE)	Parametric (FDR)	Parametric (Monte Carlo)	Permutation	Unknown	
Voxelwise	72	18		2		92
Clusterwise	155	12	92	6	5	270
TFCE				9		9

*There were 17 studies reporting uncorrected statistics; thus, only 371 studies were included in the table. TFCE, threshold-free cluster enhancement.*

### Cluster-Defining Primary Threshold (CDT) of Studies Using the Clusterwise Inferential Method

As mentioned above, 270 studies used clusterwise inference and thus required a CDT. Nearly half of them (128, 47.4%) defined their CDTs at or more stringent than *p* = 0.001 (**Table 5**). For studies using SPM, AFNI, and FSL, the proportions of CDTs reaching this standard were 70.9%, 37.6%, and 23.1%, respectively (**Figure 1**). Eighteen studies (6.7%) did not report their CDTs. The CDT level did not have a significant correlation with the sample size (*r*^2^ = 0.001, *p* = 0.683). One of the studies had a sample size of 1,299 subjects, which was much larger than the second-largest sample size at 429. If this outlier was excluded, there was still no significant correlation (*r*^2^ = 0.007, *p* = 0.180).

**Table 5:** Cluster-defining primary thresholds (CDTs) of 270 studies using clusterwise inferences.

CDT (*p*-value)	*N*	%
0.05	9	3.3
0.025	1	0.4
0.02	1	0.4
0.0125	1	0.4
0.01	60	22.2
0.005	49	18.1
0.001	124	45.9
0.0004	1	0.4
0.0001	2	0.7
0.00001	1	0.4
Unknown	21	7.8

## Discussion

The updated literature survey reported in this study reaffirmed that clusterwise inference remains the mainstream approach (270/388, 69.6%) for a cohort of 388 fMRI studies, compared to the previous numbers reported by Carp (2012) (53.2%), Guo et al. (2014) (63%), and Woo et al. (2014) (75%). There were still around 53% (142/270) studies using clusterwise inference that chose a more liberal CDT than *p* = 0.001 (*n* = 121) or did not report their CDT (*n* = 21), down from around 61% reported in Woo et al. (2014). The ratio of studies reporting uncorrected statistics was much lower than the ratios reported by Carp (2012) (40.9%), Guo et al. (2014) (19%), and Woo et al. (2014) (6%).

With regard to the sample size used in the surveyed studies, the median sample size was 33. A previous study reported that the median sample size used in the studies published in 2015 was 28.5, based on automated data extraction from Neurosynth ^1^ database (Poldrack et al., 2017). It was reassuring that studies using clusterwise inference with smaller sample sizes did not use more liberal CDTs.

In terms of inferential methods, it is still true that FSL studies mainly set their CDTs at *p* = 0.01 (default setting of the software), which is more liberal than the *p* = 0.001 that was highly recommended by various reports (Woo et al., 2014; Eklund et al., 2016; Roiser et al., 2016). Compared with the articles surveyed by Woo et al. (2014), a similar proportion of FSL studies surveyed in the current report used *p* = 0.001 or more stringent thresholds (around 23.1% vs. 20%). The false-positive rate may be influenced by multiple factors, such as the degree of spatial smoothing, experiment paradigm, statistical test performed and algorithms written in the statistical software. Hence, even if the statistical thresholds were set according to recommendations, the rate of false positives could still be high and inhomogeneous across the brain (Eklund et al., 2016). Therefore, some may advocate the use of false-discovery rate (FDR) (Genovese et al., 2002) or non-parametric approaches (Nichols and Holmes, 2002). However, few studies used FDR or non-parametric methods. Potential drawbacks of these methods are that problems may arise when inference is drawn from non-parametric methods (Hupé, 2015), whereas FDR results depend on the probability of non-null effects, which conceptually may not always be valid and different studies may set different thresholds (Hupé, 2015). Regardless of the theoretical methods used, the effect sizes should be reported alongside the brain maps of *p*-values for better comprehension of the results (Wasserstein and Lazar, 2016).

The current study has certain limitations. It would be beneficial to evaluate the effects of altering the statistical thresholds on the outcomes of the surveyed articles. However, it is not possible for a literature survey to achieve this. It should be noticed that the statistical practice is only one of the important aspects of an article. Readers should also evaluate other aspects—such as methodological details, study power and the flexibility of the analyses. It is important for readers to notice the statistical threshold used for different parts of the results. All of these may influence the quality of an article. Publishing replication studies regardless of statistical significance may help readers better comprehend the data quality (Yeung, 2017). Meanwhile, conducting meta-analysis of functional neuroimaging data can also establish consensus on the locations of brain activation to confirm or refute hypothesis (Wager et al., 2007; Zmigrod et al., 2016; Yeung et al., 2017b,d, 2018).

## Conclusion

A considerable amount of studies still used statistical approaches that might be considered as having inadequate control over false positives. There were still around 30% SPM studies that chose a more liberal CDT than *p* = 0.01 or did not report their CDT, in spite of the present recommendations. For FSL studies, it seemed that the CDT practice had no sign of improvement since the survey by Woo et al. (2014). A few studies, as noted in **Table 5**, chose unconventional CDT such as *p* = 0.0125 or 0.004. Such practice might tend to create an impression that the threshold alterations were attempted to show “desired” clusters. As the neuroimaging literature is often highly cited and has continued to grow substantially over the years (Yeung et al., 2017a,c,e), there is a need to enforce a high standard of statistical control over false positives. Meanwhile, the median sample size of the analyzed articles did not differ largely from that of previous surveys, and studies with smaller sample sizes did not use more liberal statistical thresholds. In short, there seemed to be no change in the statistical practice compared to the early 2010s.

## Author Contributions

AY is responsible for all parts of the work.

## Conflict of Interest Statement

The author declares that the research was conducted in the absence of any commercial or financial relationships that could be construed as a potential conflict of interest.

## Footnotes

1 neurosynth.org

## Supplementary Material

FILE S1 The complete lists of the 1,020 articles that were screened initially and the 388 articles that entered the analyses.
